# A Bi_2_WO_6_/Ag_2_S/ZnS *Z*-scheme heterojunction photocatalyst with enhanced visible-light photoactivity towards the degradation of multiple dye pollutants†

**DOI:** 10.1039/c9ra05372g

**Published:** 2019-09-23

**Authors:** Soleiman Mosleh, Kheibar Dashtian, Mehrorang Ghaedi, Maryam Amiri

**Affiliations:** Department of Gas and Petroleum, Yasouj University Gachsaran 75918-74831 Iran; Chemistry Department, Yasouj University Yasouj 75918-74831 Iran m_ghaedi@yu.ac.ir +98-74-33223048 +98-74-33223048

## Abstract

A novel visible-light-driven *Z*-scheme heterojunction, Bi_2_WO_6_/Ag_2_S/ZnS, was synthesized and its photocatalytic activity was evaluated for the treatment of a binary mixture of dyes, and its physicochemical properties were characterized using FT-IR, XRD, DRS and FE-SEM techniques. The Bi_2_WO_6_/Ag_2_S/ZnS *Z*-scheme heterojunctions not only facilitate the charge separation and transfer, but also maintain the redox ability of their components. The superior photocatalytic activity demonstrated by the *Z*-scheme Bi_2_WO_6_/Ag_2_S/ZnS attributes its unique properties such as the rapid generation of electron–hole pairs, slow recombination rate, and narrow bandgap. The performance of the Bi_2_WO_6_/Ag_2_S/ZnS was evaluated for the simultaneous degradation of methyl green (MG) and auramine-O (AO) dyes, while the influences of the initial MG concentration (4–12 mg L^−1^), initial AO concentration (2–6 mg L^−1^), pH (3–9), irradiation time (60–120 min) and photocatalyst dosage (0.008–0.016 g L^−1^) were investigated through the response surface methodology. The desirability function approach was applied to optimize the process and results revealed that maximum photocatalytic degradation efficiency was obtained at optimum conditions including 6.08 mg L^−1^ of initial MG concentration, 4.04 mg L^−1^ of initial AO concentration, 7.25 of pH, 90.58 min of irradiation time and 0.013 g L^−1^ of photocatalyst dosage. In addition, a possible photocatalytic mechanism of the Bi_2_WO_6_/Ag_2_S/ZnS heterojunction was proposed based on the photoinduced charge carriers.

## Abstract 2

A *Z*-scheme Bi_2_WO_6_/Ag_2_S/ZnS heterojunction was successfully synthesized as a novel visible-light-driven photocatalyst for the degradation of multiple dye pollutants.

## Graphical abstract

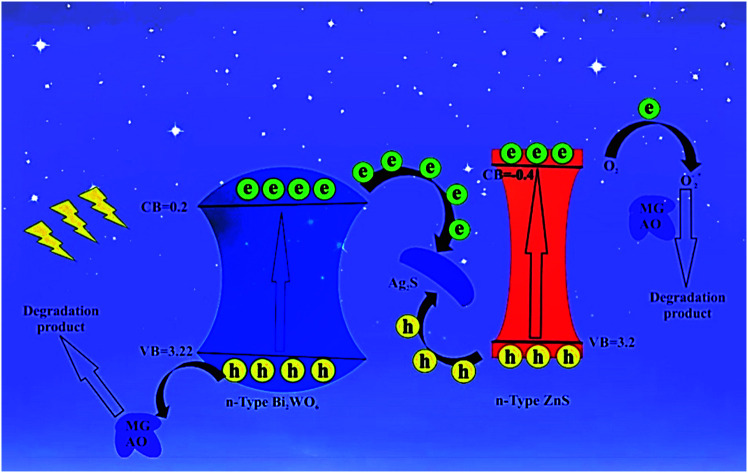

## 1. Introduction

Over the past decades, the untreated discharge of organic dyes from various industries such as textiles, dying, tanner, pulp and paper, paint and pigments has created huge risks to the environment; over 15% of the total quantity (greater than 700 000 metric tons) of dyes produced annually is lost during manufacturing and pollutes the environment.^1–3^ Many organic dyes such as auramine-O (AO) and methyl green (MG) can have adverse impacts on both aquatic organisms and human beings. The presence of such compounds in water can lead to mutagenicity, carcinogenicity, and the dysfunction of the kidneys, liver, brain, reproductive system and central nervous system in human beings.^4^ Auramine-O is a basic dye that is used to dye wool, silk, acrylic fibers, and leather in the textile industries; however its presence in water source causes acute oral toxicity, carcinogenicity, cytotoxicity, DNA damage, genotoxicity, and mutagenicity.^5^ Furthermore, triphenylmethane dyes, which are used as colorants and antimicrobial agents in diverse industries, are also known as organic water pollutants.^6^ Triphenylmethane dyes are used extensively in the textile industry for dyeing nylon, wool, cotton, and silk, as well as for coloring oil, fats, waxes, varnish, and plastics.^7^ Methyl green is a triphenylmethane-type dicationic dye that is used for the staining of solutions in medicine and biology.^8^ The presence of MG in the water resources is a great concern due to its potential toxicity to the ecosystem. Accordingly, in the present work, MG and AO dyes were selected as the target pollutants for degradation using a photocatalytic process. Since the conventional treatment processes, including biodegradation, adsorption, flocculation–coagulation, electro-coagulation and ion exchange, suffer from some drawbacks such as insufficient efficiency, requirements of the post-separation process, and creation of secondary pollutants, the photocatalytic degradation process was chosen as a promising approach for the simultaneous treatment of MG and AO dyes.^9–11^

In the past decades, photocatalytic degradation has attracted much attention for the treatment of various organic contaminants such as pesticides, dyes, textile effluents and other complex compounds.^12,13^ In this framework, visible-light-driven semiconductors have been regarded as the most promising materials for constructing the photocatalysts. Unfortunately, conventional semiconductors suffer from some serious disadvantages such as low surface area, low chemical stability, fast photo-generated electron–hole pair recombination, and insufficient reusability, which seriously hinder their further utilization in practical wastewater treatment.^14,15^

Amongst the various procedures such as morphology control, heterojunction semiconductor construction, element doping or noble metal deposition, which have been proposed to overcome the drawbacks of conventional photocatalysts, the construction of heterojunctions has been proven to be an effective technique.^16^ Accordingly, different methods, including sol–gel synthesis, ball milling, hydrothermal synthesis, and co-precipitation, have been used to fabricate heterojunction photocatalysts.^17,18^ Although a great deal of effort has been made to exploit more efficient heterojunction photocatalysts over the past few decades, the rapid recombination of photo-induced electron–hole pairs and sluggish charge transport is still an unsolved challenge.^19^ In this regard, the present work focuses on the construction of an efficient visible-light-driven heterojunction photocatalyst to overcome the obstacles of rapid charge carrier recombination.

The Bi-based photocatalysts such as BiOI, BiVO_4_, BiOBr, BOI, Bi_2_O_4_, and Bi_2_WO_6_ have shown good photocatalytic activity under visible light irradiation; among them, Bi_2_WO_6_ has attracted much attention for the degradation of various organic pollutants.^20,21^ Bi_2_WO_6_ is a simple aurivillius oxide with perovskite-like slabs of WO_4_^2−^ and Bi_2_O_2_^2+^, and it is a promising photocatalyst due to its non-toxicity and strong oxidizing power.^22^ Nevertheless, the photocatalytic activity of pure Bi_2_WO_6_ is still not satisfactory due to its weak migration efficiency of electron–hole pairs, and weak surface adsorption properties.^23^ Since the bare Bi_2_WO_6_ does not have enough photocatalytic activity, different hierarchical architectures and surface modifications were applied to enhance its photocatalytic activity performance, including nanoplates, porous thin films, 3D nest like mesoporous architectures, and flower sphere like complex structures.^24^ The bare Bi_2_WO_6_ has two major drawbacks: firstly, the absorption edge of pure Bi_2_WO_6_ is *ca.*, 450 nm which overlaps a small part of the solar spectrum leading to the unsatisfactory photo-response range; secondly, the fast recombination rate of photoinduced charge carriers leads to low quantum efficiency due to the short lifetimes of the electron–hole pairs.^25^ The coupling of bare Bi_2_WO_6_ with other semiconductors could be an effective strategy to enhance its photocatalytic performance during the promotion of the photoinduced charge carrier separation, and the expansion of the visible light responsive range.^12,26^ The co-modified Bi_2_WO_6_ could overcome the common drawbacks of the pure photocatalyst and enhance the charge-separation efficiency, and in this regard, the *Z*-scheme heterojunction construction is a promising approach.^27,28^ The enhanced charge transfer in the *Z*-scheme photocatalyst corresponds to the interface defects and the *Z*-scheme (noble metals, such as Au, Pt and Ag), where the transfer of electrons *via* the *Z*-scheme was more feasible as it relates to the superior conductivity of metals.^29^ Accordingly, metallic Ag is a promising choice for *Z*-scheme heterojunctions amongst the noble metals. Different studies have investigated the effects of metallic Ag on the enhanced photocatalytic activity of the Ag–Bi-based heterojunctions.^30–32^ The addition of Ag as a noble metal to Bi-based photocatalysts can boost the photo-electrochemical properties and introduce a new opportunity to overcome the low charge separation efficiency.^33^ Investigations have revealed that metallic Ag could improve the transfer capability of photo-generated electron–hole pairs of the Ag_3_PO_4_/BiVO_4_ composite.^34^ Furthermore, investigations indicated that metallic Ag has an undeniable role in the charge separation promotion and photoactivity enhancement for the construction of ternary heterojunctions including Ag@g-C_3_N_4_@BiVO_4_ [5], Ag/Ag_2_CO_3_/BiVO_4_,^35^ Co_3_O_4_/Ag/Bi_2_WO_6_,^36^ CuWO_4_/Ag/BiOI,^21^ and Ag–AgI/BiOI–Bi_2_O_3_.^37^ These heterojunction photocatalysts enhance the photo-excited carrier separation related to the undeniable role of Ag, which can induce localized surface plasmon resonance. Consequently, these Ag–Bi-based photocatalysts not only improve the rate of the electron–hole pair generation but also expand the visible light-responsive range.

Ag_2_S as a low band gap cocatalyst is a promising choice for a visible-light-driven photocatalyst.^38^ Besides, Ag_2_S not only has a high absorption coefficient but also is free from toxic heavy elements like Pb and Cd and thus possesses negligible toxicity as compared to other narrow band gap photocatalysts.^39^ The high stability and excellent optical limiting properties together with the abovementioned characteristics allow Ag_2_S to be an appropriate choice to enhance the Bi_2_WO_6_ photocatalytic activity.^40^

Metal sulfide semiconductors have attracted considerable attention for photocatalytic degradation owing to their exceptional chemical and physical properties.^41^ Among these, zinc sulfide (ZnS) is a low-cost, non-toxic and naturally abundant semiconductor material that has good photocatalytic activity due to the rapid generation of electron–hole pairs.^42^ Despite the rapid generation of electron–hole pairs induced by photoexcitation, poor photon absorption under visible light irradiation hinders the practical application of ZnS.^43,44^

Herein, we report a novel *Z*-scheme heterojunction, Bi_2_WO_6_/Ag_2_S/ZnS, to improve the photocatalytic degradation efficiency of pollutant dyes. The Bi_2_WO_6_/Ag_2_S/ZnS *Z*-scheme heterojunction not only facilitates the charge separation and transfer but also maintains the redox ability of its components.^45^ The *Z*-scheme Bi_2_WO_6_/Ag_2_S/ZnS is an efficient heterojunction photocatalyst owing to the unique properties of metallic Bi such as low cost, highly anisotropic Fermi surface, low carrier density, long mean free path of the carrier, and small band. The experiments were carried out using central composite design (CCD) based on the response surface methodology (RSM), while the desirability function approach was applied for process optimization. The analysis of variance (ANOVA), a powerful mathematical and statistical technique, was applied to provide a quadratic model for the prediction of the degradation efficiency. Finally, the degradation mechanism was studied and discussed graphically and theoretically to describe the enhanced photocatalytic activity based on the photoinduced charge carriers.

## 2. Experimental

### 2.1. Material and reagents

Bismuth nitrate pentahydrate (Bi(NO_3_)_3_·5H_2_O), sodium tungstate dihydrate (Na_2_WO_4_·2H_2_O), copper nitrate trihydrate (Cu(NO_3_)_2_·3H_2_O), polyvinylpyrrolidone (PVP, MW 40 000), nickel nitrate tetrahydrate (Ni(NO_3_)_2_·4H_2_O), HCl, NaOH, auramine-O (AO) and methyl green (MG) were purchased from Merck company (Darmstadt Germany). All reagents were analytical grade and used without any further purification, while deionized (DI) water was used for all experiments.

### 2.2. Instrumentation

The absorbance of a binary mixture of dyes including MG and AO was measured using a UV-vis spectrophotometer (model PG 180+ instrument, England). The pH of the solution was adjusted using dilute HCl or NaOH and measured using a pH-meter (Metrohm 691 pH meter, Switzerland). A Hermle Labortechnik GmbH centrifuge model Z206A (Germany) was used to accelerate the phase separation. The morphologies of photocatalyst samples were investigated using field emission scanning electron microscopy (SEM: T3 Tescan) under an acceleration voltage of 26.00 kV. X-ray diffraction (XRD, Philips PW 1880, Philips, Amsterdam, Netherland) was applied to characterize the phase and structure of the photocatalysts using CuKα radiation (40 kV and 40 mA). An ultrasonic bath with a heating system (Tecno-GAZ SPA Ultra Sonic System) at 40 kHz frequency and power of 130 W was used for the ultrasound-assisted synthesis procedure. The FT-IR spectra of compounds were recorded on an FT/IR-680 instrument (JASCO-Japan) in the range of 400–4000 cm^−1^ using KBr pellets with sample to KBr ratio of 1 : 100. Other equipment, software and chemical reagents were used according to manufacturer recommendations similar to our previous publications.^46–48^

### 2.3. Synthesis procedure

In a typical n-type Bi_2_WO_6_ synthesis, 1.0 mmol of Bi(NO_3_)_3_·5H_2_O and 1.0 g of PVP were added to a mixture of 35 mL of DI water, ethanol, and acetic acid in a 3 : 1 : 1 ratio and then subjected to vigorous mixing for 20 min under ultrasonic irradiation. Then, 0.5 mmol of sodium tungstate in 5 mL of DI water was added to the abovementioned clear solution and sonicated for 30 min. The resulting solution was then placed in a Teflon-lined stainless steel autoclave and heated in an oven at 180 °C for 12 h. Next, the samples were cooled to room temperature, washed sequentially with ethanol and DI water, three times each, and then dried in an oven at 70 °C for 24 h.

The synthesis of Bi_2_WO_6_/Ag_2_S/ZnS was carried out as follows: 0.75 g of the synthesized Bi_2_WO_6_ and 0.2 g of PVP were mixed in 100 mL of DI water, and then sonicated for 30 min. Afterwards, 0.24 g of Na_2_S·2H_2_O was added to above mixture and then stirred for 1.0 hour in the presence of ultrasound waves. Then, 0.1 g of AgNO_3_ and 0.25 g of Zn (NO_3_)_2_·6H_2_O were added to the mixture, and the obtained black suspension was dispersed in the presence of ultrasound waves for 30 min. The resulting mixture was placed in a stainless steel autoclave containing a Teflon vessel, and the autoclave was placed inside an oven at 120 °C. Finally, the autoclave was cooled slowly and the obtained sediment was separated using centrifugation, then washed several times with a mixture of water and ethanol solvent (1 : 3) and dried inside an oven at 60 °C. To compare the degradation rate as well as the energy gap calculations of each compound, Bi_2_WO_6_/Ag_2_S, and Bi_2_WO_6_/ZnS compounds were prepared separately, and in the absence of ZnS and Ag_2_S reagents, respectively.

### 2.4. Sequential experimental design

The process modeling was carried out through the CCD-RSM technique, which provides a valid map of the experimental domain.^49,50^ RSM as a cost-effective technique evaluates the effect of significant operational parameters on the response with at least experimental runs.^51^ The consumption of less reagent and material, reduction of systematic error, and time-saving are the benefits of the CCD-RSM technique.^52^ The influence of five operational parameters including pH (A), photocatalyst dosage (B), irradiation time (C), initial concentration of MG (D) and initial concentration of AO (E) on the degradation efficiency was evaluated using small CCD composed of 26 experiments (Table S1†). The significance of the operational parameters and their interactions were studied through the analysis of variance (ANOVA) based on the *P*-values, *F*-values and non-significant lack of fit, while RSM was applied to providing a quadratic model (eqn (1)) in terms of operational parameters to predict the optimum process conditions as follows:^53,54^1
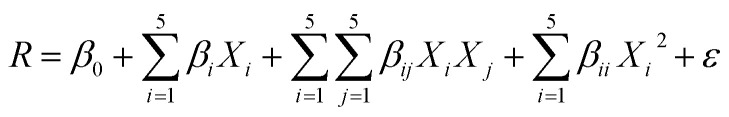
where *R* represents the predicted photocatalytic degradation percentage as the response, while *X*_*i*_ and *X*_*j*_ are the independent parameters, *ε* is the residual term and *β*_0_, *β*_*i*_, *β*_*ii*_ and *β*_*ij*_ are the model constant, linear coefficient, quadratic coefficient and the cross-product coefficient, respectively.

### 2.5. Photocatalytic degradation process

Each experimental run was performed based on the CCD-RSM technique, and certain amounts of the Bi_2_WO_6_/Ag_2_S/ZnS photocatalyst were added to the mixture of MG and AO dyes solution with specified initial concentrations, followed by mixing. HCl and/or NaOH solutions were used to adjust the pH of the solution. After transferring the above-prepared solution into the reactor tank, before switching the light source, it was kept in the dark condition until the adsorption–desorption equilibrium was reached. The degradation efficiency during the photocatalytic process was estimated as follows:2
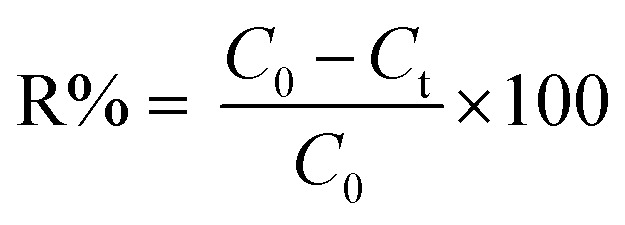
where *R* is the photocatalytic degradation efficiency (%), while *C*_0_ (mg L^−1^) and *C*_t_ (mg L^−1^) are the initial and residual concentrations of dyes in solution, respectively.

## 3. Results and discussion

### 3.1. Characterization of the photocatalyst

**Fig. 1:**
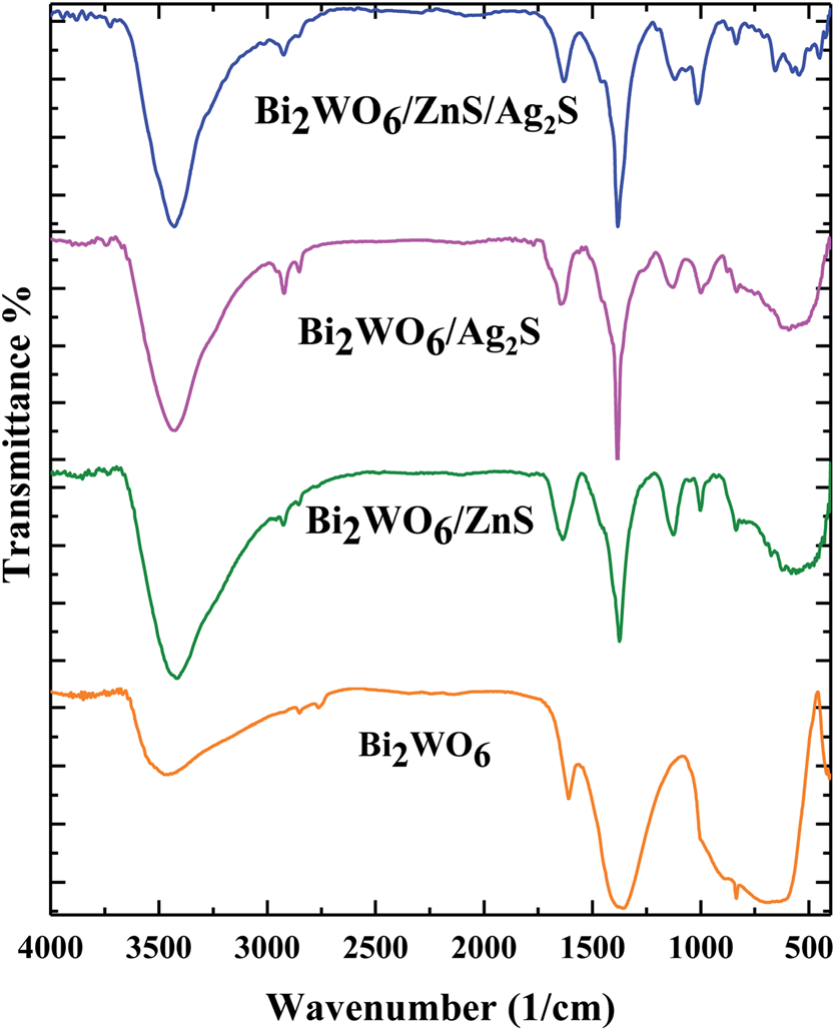
FTIR spectra of Bi_2_WO_6_, Bi_2_WO_6_/Ag_2_S, Bi_2_WO_6_/ZnS, and Bi_2_WO_6_/Ag_2_S/ZnS.

FT-IR spectra (Fig. 1) were carried out to investigate the presence of the functional groups. The typical peaks located at 3300 cm^−1^, 2930 cm^−1^, 1650 cm^−1^, 1450 cm^−1^, 890 cm^−1^ and 570 cm^−1^ are attributed to the –OH vibration of the adsorbed H_2_O on the surface of the photocatalysts, C–H asymmetric stretching of polyvinylpyrrolidone, O–H asymmetric bending, W–O–W symmetric stretching, Bi–O and W–O asymmetric stretching, W–O symmetric stretching, respectively (Fig. 1).

The FT-IR spectra related to the Bi_2_WO_6_/ZnS, and Bi_2_WO_6_/Ag_2_S clearly show the M–S (M = Ag, Zn) symmetric stretching vibration at 1000 cm^−1^, while the symmetrical stretching vibration attributed to the S–H band was observed at 980 cm^−1^ for both samples. All of the bands mentioned are visible in the final Bi_2_WO_6_/Ag_2_S/ZnS composition spectra.

**Fig. 2:**
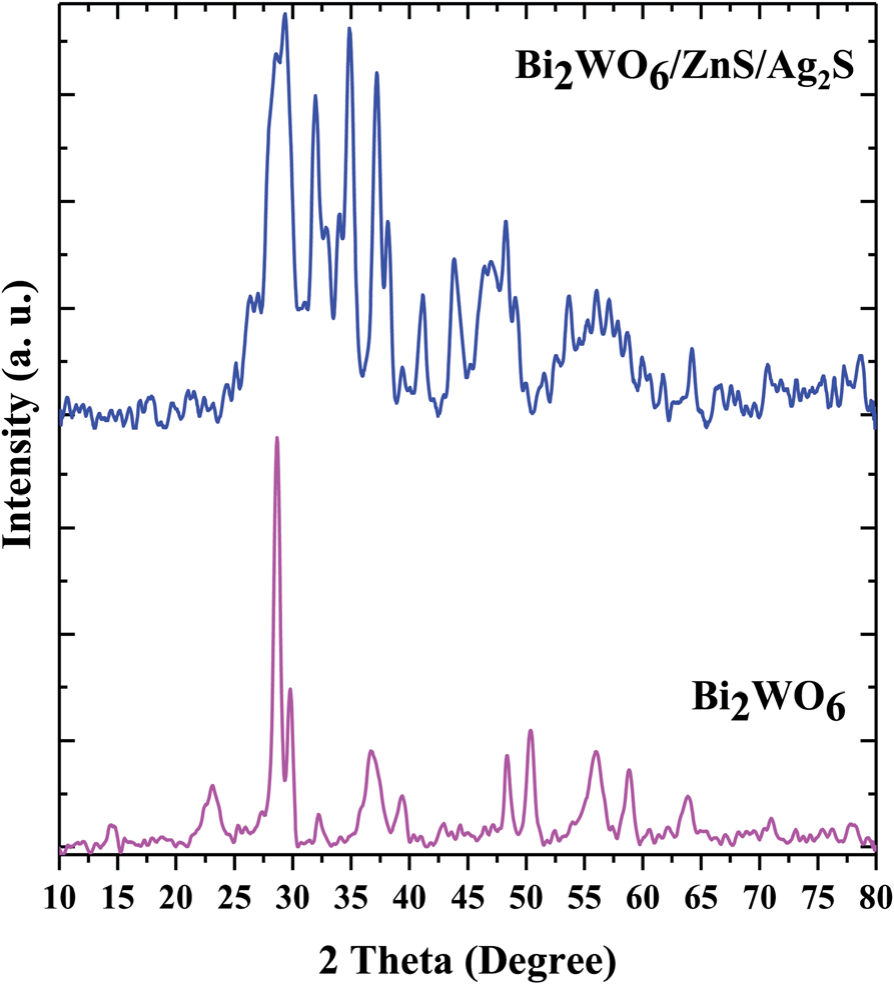
X-ray diffraction patterns of Bi_2_WO_6_ and the Bi_2_WO_6_/Ag_2_S/ZnS heterostructure.

The structural characteristics and phase composition of Bi_2_WO_6_ and Bi_2_WO_6_/Ag_2_S/ZnS were studied by XRD (Fig. 2). The peaks corresponding to the Bi_2_WO_6_, Ag_2_S, and ZnS compounds are marked with circles, squares, and triangles, respectively. The diffraction peaks of the pure Bi_2_WO_6_ sample are well-matched with the standard references corresponding to the reference code 96-901-1800. The peaks crystalline plates of Ag_2_S are well suited to the Ag_2_S-β monoclinic structure with reference code of 96-101-1338, while ZnS crystalline plates with a cube-like structure conform to the reference code of 96-110-0045.

**Fig. 3:**
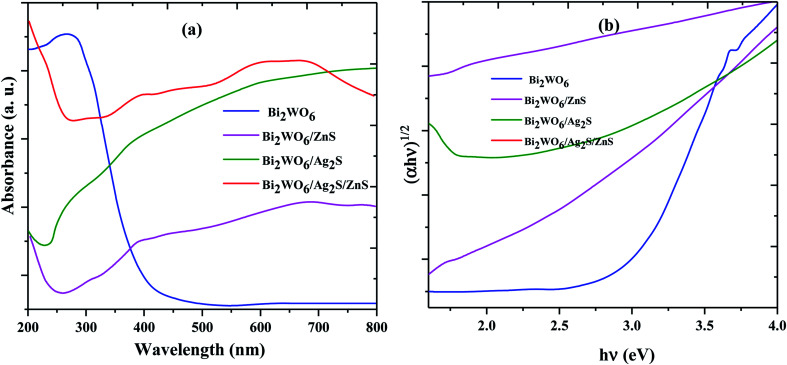
UV-vis DRS (a) and the plots of (*αhθ*)^2^*versus* photon energy for the band gap energies (b) of prepared samples.

The UV-visible absorption spectra related to the Bi_2_WO_6_, Bi_2_WO_6_/Ag_2_S, Bi_2_WO_6_/ZnS, and Bi_2_WO_6_/Ag_2_S/ZnS are shown in Fig. 3. The effect of the samples' energy gap was obtained using the tangent plot of the modified Kubelka–Munk function *versus* the photon energy on the horizontal axis. The energy gap of the Bi_2_WO_6_ and Bi_2_WO_6_/ZnS was estimated at 2.76 eV and 1.8 eV, respectively, which indicated their prominent potential for a visible-light-driven photocatalytic degradation process.

To study the photoexcited charge carrier mechanism, the potentials of the conduction band (CB) and valence band (VB) edges of the as-prepared materials were evaluated by Mulliken electronegativity theory: 
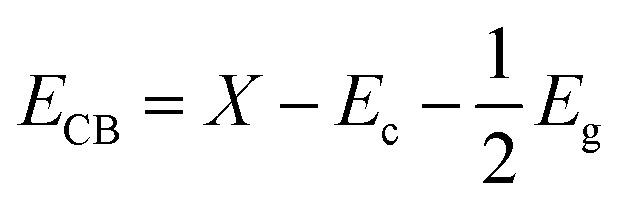
, 0.5 (*E*_g_). *X* is the absolute electronegativity of the atom semiconductor, *E*_C_ is the energy of free electrons with the hydrogen scale (4.5 eV); *E*_g_ is the band gap of the semiconductor. Therefore, the CB values related to Bi_2_WO_6_, Ag_2_S and ZnS were found to be 0.2 eV, −0.45 eV and −0.4 eV, respectively (Fig. 10). The VB can then be determined by *E*_VB_ = *E*_CB_ + *E*_g_, and the values are 0.6 eV, 3.22 eV and 3.2 eV for Bi_2_WO_6_, Ag_2_S and ZnS, respectively.

**Fig. 4:**
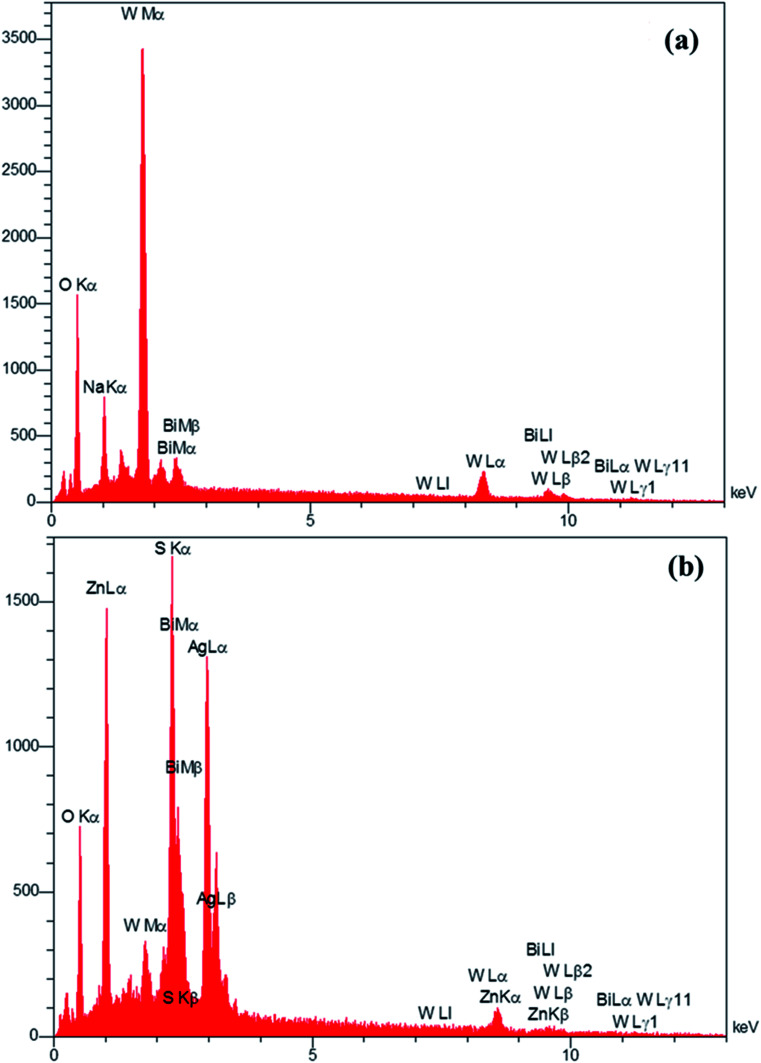
EDS pattern of Bi_2_WO_6_ and Bi_2_WO_6_/Ag_2_S/ZnS samples.

The EDS analysis was used to determine the elements in the prepared Bi_2_WO_6_/Ag_2_S/ZnS composition (Fig. 4). The presence of the Bi, O, W and impure Na (exist in the precursor material) for the Bi_2_WO_6_ compound (Fig. 4a), and the presence of Ag, Zn, S, Bi, O and W elements in the Bi_2_WO_6_/Ag_2_S/ZnS sample (Fig. 4b) confirmed the successful preparation of the desired compounds.

**Fig. 5:**
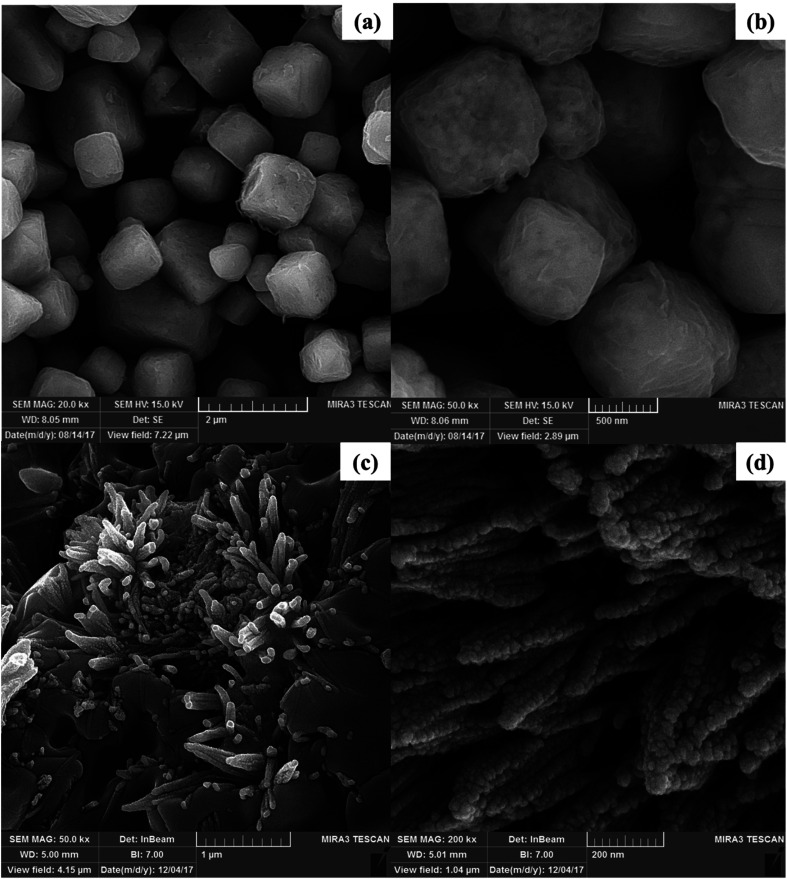
FE-SEM images of Bi_2_WO_6_ in two magnifications (a and b) and Bi_2_WO_6_/Ag_2_S/ZnS (c and d).

The morphology and size of the synthesized nanoparticles were investigated using FE-SEM analysis (Fig. 5). The FE-SEM images of the Bi_2_WO_6_/Ag_2_S/ZnS composite well indicate the growth of the nano-rods on the Bi_2_WO_6_ surface which is made of the spherical Ag_2_S/ZnS nanoparticles (Fig. 5c and d). The growth of the nano-rods on the Bi_2_WO_6_ surface makes it easier to access the photocatalyst surface which causes more interaction between dyes and photocatalyst surface particles.

### 3.2. Analysis of variance (ANOVA)

The significance of the quadratic model was evaluated through the ANOVA technique based on the *F*-value, *P*-value and lack of fit tests. The fit summary extracted from the CCD-RSM was used to select the appropriate model. The first part of the fit summary includes the sequential model sum of squares, which evaluates the mean square, degree of freedom, *F*-value test as well as *P*-value test for quadratic models (Tables S2 and S3†). The second part evaluates the significance of the proposed model based on the lack of fit (LOF) contribution. LOF shows the level of non-conformance of the data, while its *P*-value should be more than 0.05 (Table S4†). In the third part, the proposed model is evaluated based on a statistical summary of the model (Table S5†) such as regression correlation squared (*R*^2^), adjusted *R*-squared (*R*_adj._^2^), predicted *R*-squared (*R*_pred._^2^) and the predicted residual error sum of squares (PRESS).

Finally, the following equations were obtained based on the ANOVA for the prediction of MG and AO photocatalytic degradation percent during the process, respectively:

3 *R*_MG_% = 73.70 + 2.37*A* + 6.42*B* + 3.71*C* − 11.13*D* − 6.77*E* − 10.84*AB* − 9.28*AC* + 1.69*AD* + 9.12*AE* − 6.25*BC* + 6.28*BD* + 8.57*BE* − 6.80*CE* + 8.25*DE* + 1.133*A*^2^

4 *R*_AO_% = 65.66 + 1.92*A* + 7.93*B* + 5.29*C* − 7.17*D* − 4.68*E* + 2.01*AB* + 2.01*AC* + 0.71*AD* + 0.70*AE* − 1.55*BD* − 1.55*BE* − 1.55*CD* − 8.25*CE* − 1.55*DE* + 0.91*A*^2^

where *R*_MG_% and *R*_AO_% are the photocatalytic degradation percentages of MG and AO, respectively. The negative coefficients related to each term in the quadratic equations indicate the unfavorable effect on the degradation efficiency, while the positive coefficients indicate a favorable effect.

The *P*-value related to the lack of fit for each model term should be higher than 0.05, which confirms the possibility of low noise (Table S4†). Other evaluations including the adequate precision, standard error, coefficient variation%, mean and PRESS indicate the conformability of the selected model for the degradation process of AO and MG (Table S5†).

The quadratic regression model for the prediction of the MG and AO degradation efficiency was highly significant, while the model *F*-value with very low probability value (*P*-value < 0.0001) revealed the high significance of the quadratic model (Tables S2–S4†). Furthermore, the statistical coefficient of determination of *R*-squared (0.99 for both AO and MG), adjusted *R*-squared (0.99 for both AO and MG) as well as predicted *R*-squared (0.89 for AO and 0.82 for MG) with their high values indicated that the obtained model has good performance for the prediction of the degradation efficiency (Table S5†).

### 3.3. Response surface graphs

**Fig. 6:**
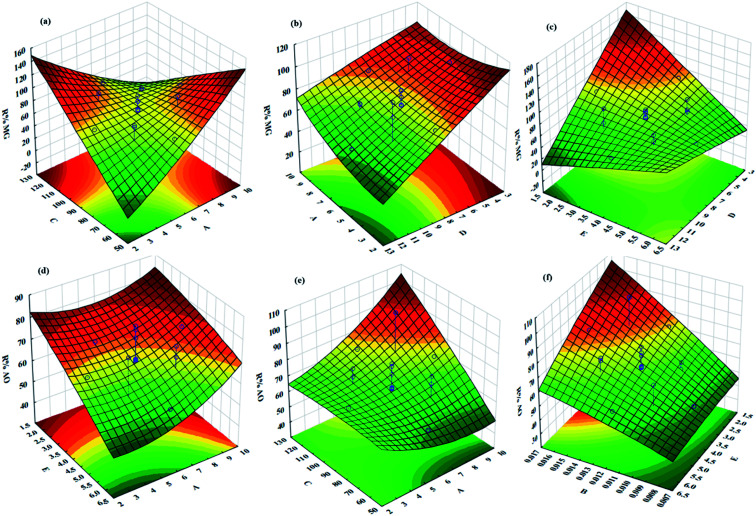
3D plots of RSM at optimal conditions: pH (*A* = 7.25), photocatalyst mass (*B* = 0.013 g), irradiation time (*C* = 90.58 min), initial concentration of MG (*D* = 6.08 mg L^−1^) and initial concentration of AO (*E* = 4.04 mg L^−1^).

The influences of the operational parameters and their combinational interactions on the degradation efficiency are illustrated *via* response surface graphs (Fig. 6). The influence of the initial dye concentrations on the photocatalytic degradation indicated that at higher concentrations lower efficiency was achieved due to the lower ratio of the activated photocatalyst for each dye molecule (Fig. 6). Besides, increasing the initial AO and MG concentration leads to the creation of dark conditions in the reactor vessel, which causes a reduction in the penetration depth of light. Under this condition, fewer photocatalyst sites can be activated and consequently, the degradation efficiency decreases.

The irradiation time has a positive impact on the photocatalytic degradation efficiency owing to greater exposure of dyes molecules and consequently, more contact between oxidant radicals and dye molecules, which lead to more degradation (Fig. 6). The role of pH as an undeniable factor in the photocatalytic degradation process was evaluated in the range of 3–9, while the obtained results revealed that maximum efficiency was found at pH 7.25 (Fig. 6).

Photocatalyst dosage considerably influences the photocatalytic degradation of dyes, while increasing its value causes more available active site areas for the degradation of AO and MG molecules (Fig. 6). It should be noted that exceeding the photocatalyst dosage from a specified value causes a reduction in the photocatalytic degradation efficiency due to the creation of a dark condition that diminishes the light path penetration.

### 3.4. Optimization study

**Fig. 7:**
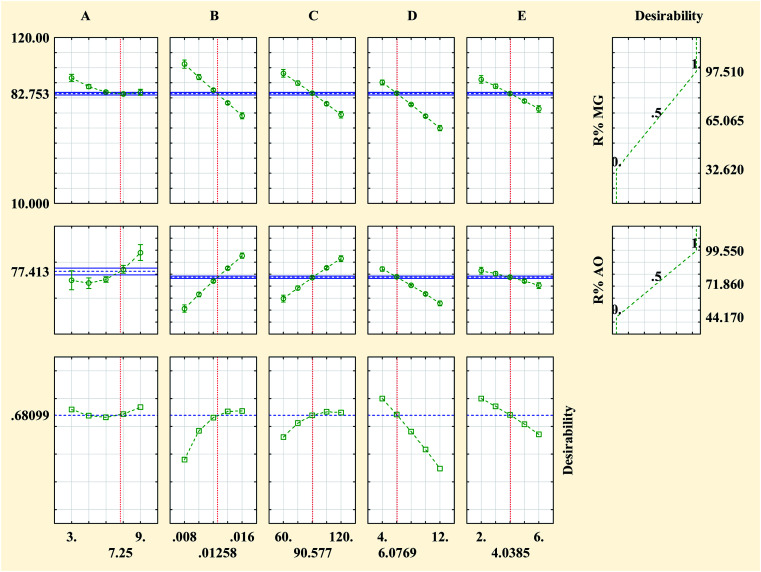
Profiles of the predicated values and desirability functions for the photocatalytic degradation process; dashed lines show optimum values.

Optimization of the photocatalytic process (Fig. 7) to provide the maximum degradation efficiency corresponding to optimal operational parameter values was performed using the STATISTICA software (ver. 10.0). The STATISTICA acts based on the desirability function (DF), which ranges from 0 (undesirable situation) to 1.0 (ideal situation). The maximum photocatalytic degradation percentage for MG and AO was found to be 82.75% and 77.41%, respectively, under the optimum conditions including pH (*A* = 7.25), photocatalyst dosage (*B* = 0.013 g L^−1^), irradiation time (*C* = 90.58 min), initial concentration of MG (*D* = 6.08 mg L^−1^) and initial concentration of AO (*E* = 4.04 mg L^−1^).

**Fig. 8:**
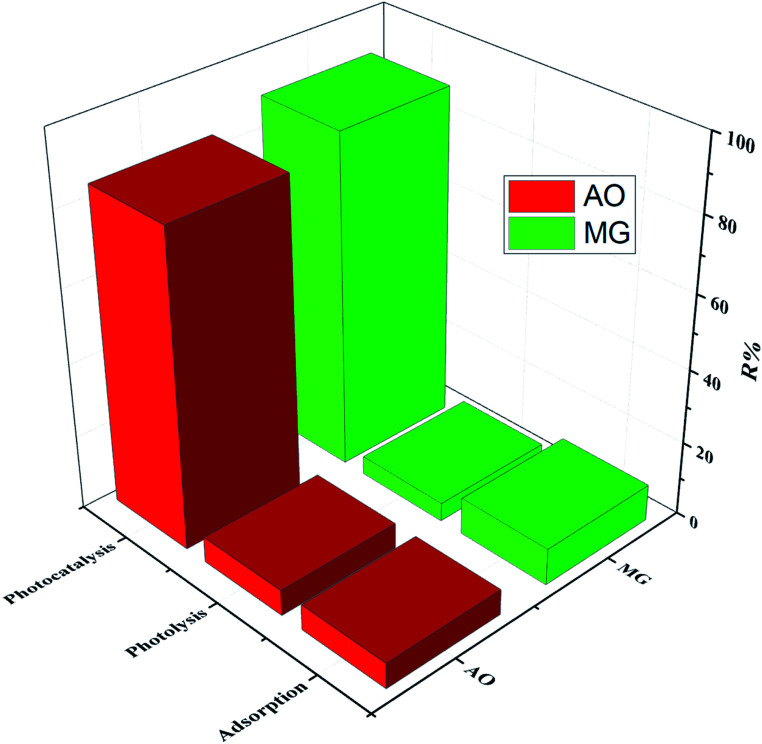
Decolonization efficiency under different processes at optimum conditions.

Furthermore, the decolonization of MG and AO dyes using different processes including adsorption (catalyst), photolysis (light irradiation without catalyst) and photocatalysis (light irradiation + catalyst) was performed at optimum conditions to evaluate the role and contribution of each process. The results indicated that decolonization percentages of dyes (MG% and AO%) by adsorption, photolysis and photocatalysis were (10.05%, 8.31%), (2.86%, 5.12%) and (82.75% and 77.41%), respectively (Fig. 8). The higher efficiency of photocatalysis rather than both adsorption and photolysis processes is related to the synergetic effect, which corresponds to the role of the simultaneous application of light irradiation and catalyst performance. In fact, the combined system has a positive effect on the degradation efficiency as compared with individual processes, while the addition of catalyst to the system in the presence of the light irradiation leads to the generation of the hydroxyl radicals, which causes more degradation of the dye structure.

## 4. Photocatalytic degradation mechanism study

**Fig. 9:**
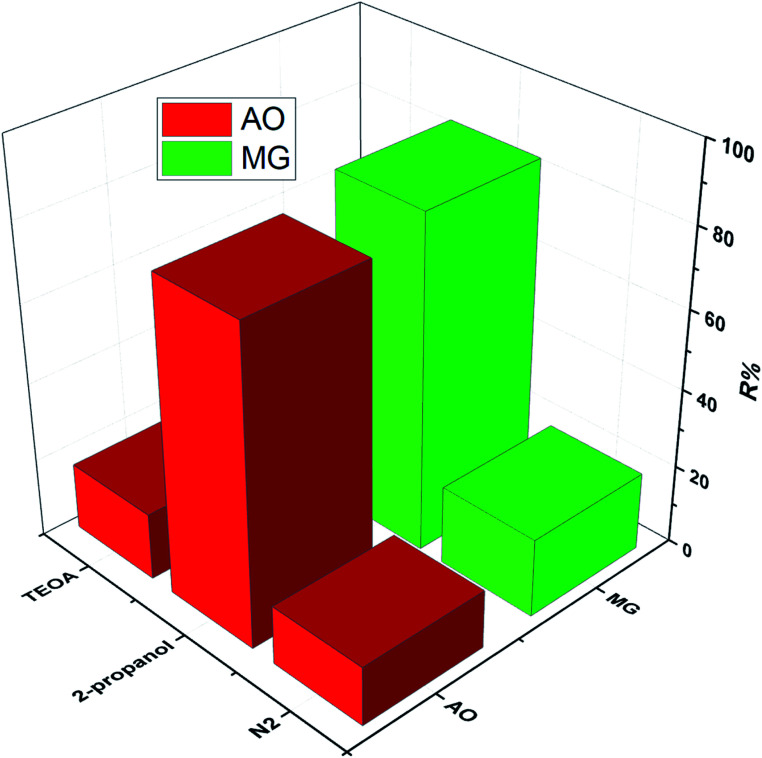
Trapping experiments for active species in the photocatalytic process.

To study the photodegradation mechanism, active species trapping experiments were performed using 2-propanol as the OH radical scavenger, N_2_-bubbling for inhibiting O_2_ radicals, and triethanolamine (TEOA) as the hole (h^+^) radical scavenger; the results are shown in Fig. 9. It can be seen that the degradation efficiencies of MG and AO were hardly affected by adding 2-propanol, indicating that the OH radical is not the main reactive species. The degradation rates of MG and AO were decreased under N_2_ atmosphere, suggesting that O_2_ plays the main role in the photodegradation process. In the presence of TEOA, the degradation rates of MG and AO were greatly inhibited, which reveals that the h^+^ was an important reactive species. As a result, it could be preliminarily concluded that the generated h^+^ and O_2_ radicals in the proposed photocatalytic system should be responsible for the enhanced photo-oxidation performance towards MG and AO degradation.^55^

**Fig. 10:**
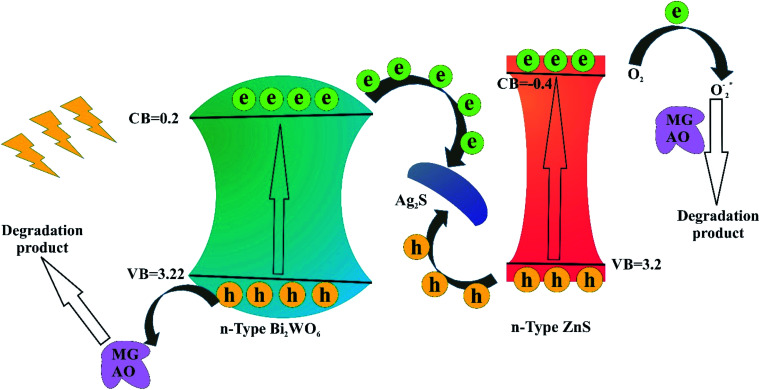
Schematic diagram of the separation and transfer of photogenerated charges in the Bi_2_WO_6_/Ag_2_S/ZnS under visible light irradiation.

Based on the results, a possible *Z*-scheme mechanism for the simultaneous photocatalytic degradation of MG and AO was proposed by Bi_2_WO_6_/Ag_2_S/ZnS (see Fig. 10). Under visible light, both Bi_2_WO_6_ and Ag_2_S/ZnS were excited; Ag_2_S, due to the low band gap, can act like a noble metal, absorb visible light, and generate electron–hole pairs.^34,56^ The electrons will be transferred to the CB of ZnS or are captured by the O_2_ in the solution to form O_2_^−^ radicals and subsequently oxidize organic substances. The remaining holes on the Ag_2_S can recombine with the excited electrons of Bi_2_WO_6_ CB due to the Schottky barrier at the metal–semiconductor interface. Because the VB of ZnS is higher than that of the Bi_2_WO_6_, the holes on the VB of ZnS can transfer to the VB of Bi_2_WO_6_ and directly oxidize organic substances that have been adsorbed in the layer of Bi_2_WO_6_, while neither oxidize OH radicals nor H_2_O into OH radicals.^29^

## 5. Conclusion

The novel Bi_2_WO_6_/Ag_2_S/ZnS was synthesized as a visible-light-driven *Z*-scheme heterojunction photocatalyst, and its performance was evaluated for the degradation of a mixture of dyes including MG and AO. The Bi_2_WO_6_/Ag_2_S/ZnS *Z*-scheme heterojunctions not only facilitate the charge separation and transfer but also maintains the redox ability of its components. The *Z*-scheme Bi_2_WO_6_/Ag_2_S/ZnS is an efficient heterojunction photocatalyst owing to the unique properties of metallic Bi such as low cost, highly anisotropic Fermi surface, low carrier density, the long mean free path of the carrier, and the small band. It is worth pointing out that the synergetic effect among Bi_2_WO_6_, Ag_2_S, and ZnS helps to improve the photocatalytic performance towards contaminant decomposition. The possible decomposition mechanism of the degradation process was studied based on the photogenerated electron transfer and charge carrier system, which indicates the high stability of the generated electron–hole pairs. The low recombination rate of electron–hole pairs meant the high performance of Bi_2_WO_6_/Ag_2_S/ZnS to achieve more photocatalytic degradation efficiency. The desirability function was used to optimize the degradation process; the results revealed that compared to pure Bi_2_WO_6_, Ag_2_S, and ZnS, Bi_2_WO_6_/Ag_2_S/ZnS has superior degradation performance with 82.75% and 77.41% for MG and AO dyes, respectively.

## Conflicts of interest

There are no conflicts to declare.

## Supplementary Material

RA-009-C9RA05372G-s001
