# Supplementary material for: A Bi2WO6/Ag2S/ZnS Z-scheme heterojunction photocatalyst with enhanced visible-light photoactivity towards the degradation of multiple dye pollutants
Source: RSC Adv. 2019 Sep 23;9(52):30100–11. doi: 10.1039/c9ra05372g (PMC9072109; doi:10.1039/c9ra05372g)
Supplement: RA-009-C9RA05372G-s001 [file RA-009-C9RA05372G-s001.pdf]

Electronic Supplementary Material (ESI) for RSC Advances.
This journal is © The Royal Society of Chemistry 2019

$Bi_2WO_6/Ag_2S/ZnS$ Z-scheme heterojunction photocatalyst with enhanced visible-light photoactivity towards multiple dye pollutants degradation

Soleiman Mosleh[a], Kheibar Dashtian[b], Mehrorang Ghaedi[b*], Maryam Amiri[b]

[a] *Department of Gas and Petroleum, Yasouj University, Gachsaran 75918-74831, Iran*

[b] *Chemistry Department, Yasouj University, Yasouj 75918-74831, Iran, Tel. /fax: +98-74-33223048*

Corresponding author:

**E-Mail: m_ghaedi@yu.ac.ir*

Table S1. Experimental factors and levels in the central composite design.

Factor	Name	Units	Minimum	Maximum	Coded	Values	Mean	
A	pH	-	3.00	9.00	4.50	7.50	6.06	
B	Photocatalyst dosage	g/L	0.008	0.016	0.010	0.014	0.012	
C	Irradiation Time	min	60.00	120.00	75.00	105.00	90.58	
D	Concentration of MG	mg/L	4.00	12.00	6.00	10.00	8.080	
E	Concentration of AO	mg/L	2.00	6.00	3.00	5.00	4.04	

Run	A	B	C	D	E	Actual R% MG	Predicted R% MG	R% AO	Predicted R% AO
1	6.0	0.008	90	8	4	61.10	60.86	49.21	48.94
2	6.0	0.016	90	8	4	86.79	86.55	80.94	80.67
3	6.0	0.012	90	12	4	51.17	50.93	50.73	50.46
4	4.5	0.014	105	6	5	79.31	79.47	76.62	76.80
5	7.5	0.014	105	6	3	66.90	67.06	99.55	99.73
6	9.0	0.012	90	8	4	83.18	82.94	73.42	73.15
7	4.5	0.010	105	10	5	32.62	32.78	46.41	46.59
8	6.0	0.012	90	8	2	86.98	86.74	74.43	74.16
9	*6.0*	*0.012*	*90*	*8*	*4*	*74.24*	*73.70*	*65.57*	*65.66*
10	4.5	0.014	75	10	5	82.83	82.99	51.69	51.87
11	6.0	0.012	60	8	4	66.01	65.77	54.49	54.22
12	*3.0*	*0.012*	*90*	*8*	*4*	*73.71*	*73.47*	*65.73*	*65.46*
13	6.0	0.012	90	8	4	73.44	73.70	65.07	65.66
14	*6.0*	*0.012*	*90*	*8*	*4*	*73.44*	*73.70*	*65.79*	*65.66*
15	4.5	0.014	105	10	3	96.08	96.24	71.63	71.81
16	6.0	0.012	90	4	4	95.71	95.47	79.41	79.14
17	*6.0*	*0.012*	*90*	*8*	*4*	*73.44*	*73.70*	*66.07*	*65.66*
18	4.5	0.010	75	6	3	81.10	81.42	59.52	59.88
19	6.0	0.012	90	8	6	59.91	59.67	55.72	55.45
20	7.5	0.010	75	10	5	72.68	72.84	44.17	44.35
21	6.0	0.012	120	8	4	80.87	80.63	75.66	75.39
22	7.5	0.014	75	10	3	54.11	54.27	69.4	69.58
23	7.5	0.014	75	6	5	97.51	97.67	74.38	74.56
24	*6.0*	*0.012*	*90*	*8*	*4*	*73.44*	*73.70*	*65.27*	*65.66*
25	7.5	0.010	105	10	3	71.62	71.78	64.12	64.30
26	7.5	0.010	105	6	5	75.41	75.57	69.1	69.28

Table S2. Sequential Model Sum of Squares for MG degradation.

R% MG						
**Sequential Model Sum of Squares**						
**Source**	**Sum of Squares**	**df**	**Mean Square**	**F Value**	**p-value Prob > F**	
Mean vs Total	1.423E+005	1	1.423E+005			
Linear vs Mean	2110.11	5	422.02	2.69	0.0515	
2FI vs Linear	3098.73	10	309.87	73.90	< 0.0001	
Quadratic vs 2FI	40.46	5	8.09	27.42	0.0012	Suggested
Cubic vs Quadratic	0.96	1	0.96	7.53	0.0517	Aliased
Residual	0.51	4	0.13			
Total	1.476E+005	26	5675.68			
**Lack of Fit Tests**						
**Source**	**Sum of Squares**	**df**	**Mean Square**	**F Value**	**p-value Prob > F**	
Linear	3140.15	16	196.26	1533.28	< 0.0001	
2FI	41.42	6	6.90	53.93	0.0009	
Quadratic	0.96	1	0.96	7.53	0.0517	Suggested
Cubic	0.000	0				Aliased
Pure Error	0.51	4	0.13			
**Model Summary Statistics**						
**Source**	**Std. Dev.**	**R-Squared**	**Adjusted R-Squared**	**Predicted R-Squared**	**PRESS**	
Linear	12.53	0.4019	0.2523	-0.1621	6101.94	
2FI	2.05	0.9920	0.9800	0.9636	191.13	
Quadratic	0.54	0.9997	0.9986	0.8966	543.16	Suggested
Cubic	0.36	0.9999	0.9994		+	Aliased

Table S3. Sequential Model Sum of Squares for AO degradation.

R% AO						
**Sequential Model Sum of Squares**						
**Source**	**Sum of Squares**	**df**	**Mean Square**	**F Value**	**p-value Prob > F**	
Mean vs Total	1.130E+005	1	1.130E+005			
Linear vs Mean	3765.24	5	753.05	179.04	< 0.0001	
2FI vs Linear	49.84	10	4.98	1.45	0.2826	
Quadratic vs 2FI	32.43	5	6.49	17.45	0.0035	Suggested
Cubic vs Quadratic	1.22	1	1.22	7.66	0.0504	Aliased
Residual	0.64	4	0.16			
Total	1.169E+005	26	4494.41			
**Lack of Fit Tests**						
**Source**	**Sum of Squares**	**df**	**Mean Square**	**F Value**	**p-value Prob > F**	
Linear	83.49	16	5.22	32.76	0.0020	
2FI	33.65	6	5.61	35.21	0.0020	
Quadratic	1.22	1	1.22	7.66	0.0504	Suggested
Cubic	0.000	0				Aliased
Pure Error	0.64	4	0.16			
**Model Summary Statistics**						
**Source**	**Std. Dev.**	**R-Squared**	**Adjusted R-Squared**	**Predicted R-Squared**	**PRESS**	
Linear	2.05	0.9781	0.9727	0.9583	160.40	
2FI	1.85	0.9911	0.9777	0.9187	312.95	
Quadratic	0.61	0.9995	0.9976	0.8212	688.15	Suggested
Cubic	0.40	0.9998	0.9990		+	Aliased

Table S4. Analysis of variance (ANOVA) results for quadratic model of degradation process.

		R% of MG					R% of	
**Source**	**df**	**Sum of Squares**	**Mean Square**	**F Value**	**p-value Prob > F**		**Sum of Squares**	**Mean Square**
Model	20	5249.30	262.47	889.35	< 0.0001		3847.51	192.38
A	*1*	*44.84*	*44.84*	*151.94*	*< 0.0001*		*29.57*	*29.57*
B	*1*	*329.99*	*329.99*	*1118.15*	*< 0.0001*		*503.40*	*503.40*
C	*1*	*110.41*	*110.41*	*374.12*	*< 0.0001*		*224.08*	*224.08*
D	*1*	*991.91*	*991.91*	*3361.04*	*< 0.0001*		*411.27*	*411.27*
E	*1*	*366.39*	*366.39*	*1241.51*	*< 0.0001*		*175.03*	*175.03*
AB	*1*	*398.60*	*398.60*	*1350.66*	*< 0.0001*		*13.74*	*13.74*
AC	*1*	*292.16*	*292.16*	*989.97*	*< 0.0001*		*13.74*	*13.74*
AD	*1*	*9.65*	*9.65*	*32.69*	*0.0023*		*1.69*	*1.69*
AE	*1*	*281.80*	*281.80*	*954.87*	*< 0.0001*		*1.68*	*1.68*
BC	*1*	*132.35*	*132.35*	*448.47*	*< 0.0001*		*0.19*	*0.19*
BD	*1*	*133.90*	*133.90*	*453.71*	*< 0.0001*		*8.11*	*8.11*
BE	*1*	*249.26*	*249.26*	*844.61*	*< 0.0001*		*8.11*	*8.11*
CD	*1*	*2.26*	*2.26*	*7.67*	*0.0394*		*8.11*	*8.11*
CE	*1*	*156.56*	*156.56*	*530.51*	*< 0.0001*		*8.11*	*8.11*
DE	*1*	*230.86*	*230.86*	*782.27*	*< 0.0001*		*8.14*	*8.14*
A^2	*1*	*34.81*	*34.81*	*117.94*	*0.0001*		*22.73*	*22.73*
B^2	*1*	*2.424E-004*	*2.424E-004*	*8.213E-004*	*0.9782*		*1.25*	*1.25*
C^2	*1*	*0.42*	*0.42*	*1.41*	*0.2886*		*1.25*	*1.25*
D^2	*1*	*0.42*	*0.42*	*1.41*	*0.2886*		*1.26*	*1.26*
E^2	*1*	*0.41*	*0.41*	*1.38*	*0.2930*		*1.25*	*1.25*
Residual	5	1.48	0.30				1.86	0.37
Lack of Fit	*1*	*0.96*	*0.96*	*7.53*	*0.0517*		*1.22*	*1.22*
Pure Error	*4*	*0.51*	*0.13*				*0.64*	*0.16*
Cor Total	25	5249.30	262.47	889.35	< 0.0001		3849.37	

Table S5. Statistical supplementary results of quadratic model.

	R% AO		R% MG
**Std. Dev.**	0.54		0.61
**Mean**	73.98		65.93
**C.V. %**	0.73		0.92
**PRESS**	543.16		688.15
**-2 Log Likelihood**	-0.81		5.18
**R-Squared**	0.9997		0.9995
**Adj R-Squared**	0.9986		0.9976
**Pred R-Squared**	0.8966		0.8212
**Adeq Precision**	132.910		101.087
**BIC**	67.61		73.60
**AICc**	272.19		278.18
